# Musical training is associated with cognitive outcomes and white matter microstructure in middle childhood

**DOI:** 10.1016/j.dcn.2026.101813

**Published:** 2026-09-06

**Authors:** Kelsie L. Lopez, Yaen Chen, Roger Burtonpatel, Lauren Raine, Arthur F. Kramer, Charles H. Hillman, Psyche Loui

**Affiliations:** a Dept. of Psychology, College of Science, Northeastern University, USA; b Biological and Medical Informatics PhD Program, University of California, San Francisco, CA, USA; c Bakar Computational Health Sciences Institute, University of California San Francisco, USA; d Dept. of Computer and Information Science, University of Pennsylvania, USA; e Dept. of Music, College of Arts, Media, and Design, Northeastern University, USA; f Dept. Of Physical Therapy, Movement, and Rehabilitation Sciences, Northeastern University, USA; g Institute for Cognitive and Brain Health, Northeastern University, USA; h Beckman Institute, University of Illinois, USA

**Keywords:** Musical training, Diffusion tensor imaging, Cognition, Academic achievement

## Abstract

Musical training is associated with improved cognitive and academic performance across development. While specific neural mechanisms have been implicated in these associations, the precise relationship between white matter structure and putative cognitive effects of musical training are unclear. The present study examined the relationships between musical training, cognitive performance, and white matter structure in middle childhood. Using a combination of behavioral assessments and diffusion tensor imaging (DTI) in a sample of 76 preadolescents (38 with musical training and 38 without musical training) between 7 and 9 years old, cognitive abilities were tested using the Woodcock-Johnson Tests of Cognitive Abilities (WJ III) and the Operation Span Task (OSPAN), while academic achievement was assessed using the Kaufman Test of Educational Achievement (KTEA II). Behavioral results revealed that participants with musical training had higher cognitive scores (*F*(5, 70) = 2.89, *p* = .02) and greater academic achievement (*F*(5, 70) = 4.09, *p* = .003) compared to those without musical training. DTI results revealed that musical training was associated with higher mode (*F*(1, 48) = 5.82, *p* = 0.02) in the right PCG to STGp tract of the arcuate fasciculus (AF), although the relationship was marginally significant following Benjamini-Hochberg correction. Together, these results provide both behavioral and neural evidence for the relationship between musical training and cognition in a sample of preadolescents.

## 1 Introduction

Childhood and preadolescence are crucial periods for neural development, where processes including synaptic refinement, white matter organization, and gray matter alterations are impacted by environmental input (Giedd et al., 1999, Petanjek and Judaš, 2011). Further, middle childhood is marked by key development in cognitive (Buttelmann and Karbach, 2017), emotional (Uhl et al., 2019), and social (Rodkin et al., 2012) domains. To aid in developmental growth, children are often encouraged to participate in engaging extracurricular activities, such as music-based activities (Simoncini and Caltabiono, 2012, Metsäpelto and Pulkkinen, 2012, Oberle et al., 2019).

Music-based extracurricular interventions during childhood have been particularly shown to improve various cognitive domains through both near and far transfer effects. Near transfer effects refer to associated outcomes that share domain-specific qualities with musical training, such as the observed effects of improved rhythmic and melodic perception (Nave-Blodgett et al., 2021, Bailey and Penhune, 2013, Morrongiello and Roes, 1990). Meanwhile, transfer effects of musical participation to non-musical skills have been observed through improved executive functioning, reading comprehension, school performance, and language skills (Rodriguez-Gomez and Talero-Gutiérrez, 2022, Corrigall and Trainor, 2011, Wetter et al., 2009, Moreno et al., 2011). For example, after 36 weeks of music lessons, children had increased test scores in cognitive ability and academic achievement compared to children who received drama lessons or no lessons (Schellenberg, 2004). In another study, children who received 20 days of music training performed better on the vocabulary domain of the Wechsler Preschool and Primary Scale of Intelligence, a measure of verbal intelligence, compared to an age-matched group who received visual art training. These improvements in verbal intelligence were positively correlated with changes in functional brain plasticity during an executive function task (Moreno et al., 2011). Finally, a 2.5 year longitudinal study in children comparing instrumental musical training with visual art training and a no-intervention group selectively found improved executive processing skills and verbal comprehension only in the music training group (Jaschke et al., 2018).

Using non-invasive neuroimaging techniques, researchers have identified specific neural mechanisms associated with musical training and cognitive function. Improvements in executive function was accompanied by greater brain activation in the supplementary motor area, anterior cingulate cortex, inferior frontal gyrus, and insula for children with musical training compared to children who participated in physical activity (Sachs et al., 2017). Another study observed improvements in phonological processing for children with dyslexia who participated in musical training, which was associated with greater bilateral activation in left-hemispheric temporoparietal regions (Zuk et al., 2018). Using a longitudinal design, Habibi et al. (2018) found evidence of higher connectivity in the corpus callosum of school-aged children who spent two years in a musical training group compared to children who were enrolled in sport training or a no activity control group.

Here, we leverage diffusion-weighted neuroimaging to uncover valuable biological information on white matter pathways in the brain associated with musical training. Diffusion-weighted neuroimaging quantifies scalars relevant for white matter microstructure, including fractional anisotropy (FA), axial diffusivity (AD), radial diffusivity (RD), and mode of anisotropy (MO) (Alexander et al., 2007). FA values reflect a geometric dimension of water diffusion within a voxel, with the shape of the tensor expected to be more anisotropic in white matter pathways compared to gray matter pathways (O’Donnell and Westin, 2011). When the tensor is more anisotropic, FA values are higher, reflecting increased myelination and predominating axon fiber axis orientation in white-matter tracts (Song et al., 2002). AD measures diffusion along the primary axis of a voxel, and decreased AD is associated with axonal injury, whereas RD reflects the average magnitude of diffusion perpendicular to the primary axis and increases with demyelination (Feldman et al., 2010). Lastly, MO distinguishes between planar crossing fibres, resulting in negative MO values, and linear fibers in one primary orientation, resulting in positive MO values (Alexander et al., 2007).

Additionally, researchers have leveraged diffusion-weighted imaging to reveal associations between language and musical training with the structure of the superior longitudinal fasciculus (SLF), which connects the parietal region with the frontal lobe. In adults, musical expertise and absolute pitch abilities was associated with left lateralization of SLF white matter fibers (Oechslin et al., 2010), and in a sample of children between 5 and 17 years old, higher FA in the left SLF was positively correlated with language abilities (Urger et al., 2015). These studies uncover a shared overlap of the SLF for language and musical participation, which likely play a role in the near and far transfer effects of musical training.

Using a subset of the cohort in the present study, AD in the left SLF was higher in children with musical training compared to children without musical training (Loui et al., 2019). While studies have investigated the role of the SLF in language and musical training, the arcuate fasciculus (AF), a component of the SLF connecting the posterior and anterior language cortices, has been particularly implicated in more specific language and music-related domains. Individuals with higher FA values in the AF demonstrate superior language learning skills (Qi et al., 2015). More specifically, FA of the left AF has been positively correlated with performance on phoneme awareness and speech perception tasks (Vandermosten et al., 2012) while FA of the right AF has been implicated in music-cued motor training (Moore et al., 2017). Further, the increased connectivity of the AF has also been found to play a major role in pitch-related grammar learning (Loui et al., 2011) and pitch perception (Loui et al., 2009). Musical training was also associated with superior speech detection accuracy in noisy environments, which was correlated with higher fractional anisotropy (FA) in the right arcuate fasciculus (AF) and lower radial diffusivity (RD) in the left anterior AF (Li et al., 2021). The structure of the AF and its associations with cognition and musical training during childhood is less known.

To evaluate the relationship of musical training, cognitive ability, and white matter microstructure within the AF, we used data collected from the FITKids2 study, a randomized control trial physical activity (PA) intervention of 7–9 year olds (Chaddock-Heyman et al., 2014, Chaddock-Heyman et al., 2018, Hillman et al., 2014). We hypothesized that children who play an instrument would exhibit higher cognitive scores and greater academic achievement in middle childhood compared to musically untrained children. Further, we hypothesized that musically trained children would exhibit differences relative to musically untrained children in white matter integrity of the arcuate fasciculus. Notably, the present study uses probabilistic tractography, a computational method that identifies individual-level white matter tracts. Probabilistic tractography allows us to better account for individual variability in anatomy than would be possible using a tract-based spatial statistics approach, which was used in a previous study with the same sample (Loui et al., 2019). Overall, our study uncovers relationships between musical training in children, white matter microstructure in the AF, and cognitive ability and academic achievement test scores to investigate the role of the AF in musical training.

## 2 Methods

### 2.1 Participants

Participants were recruited as part of the FITKids2 study at the University of Illinois at Urbana-Champaign (Chaddock-Heyman et al., 2014, Chaddock-Heyman et al., 2018, Hillman et al., 2014). In these studies, children aged 7–9 years old were recruited from East Central Illinois, USA into a randomized control trial. For the purposes of the present study, all data was drawn from the pre-intervention visit (for additional information on the intervention, see Hillman et al., 2014). Written consent was obtained from the legal guardians and written assent from the participants. Study approvals were obtained by the Institutional Review Board of the University of Illinois at Urbana-Champaign.

221 children between the ages of 7–9 years old participated in FITKids2 study. 175 participants out of the original 221 participants had complete data on musical training, as well as academic achievement and cognition scores, and thus were included in the initial sample for the present study. Children with musical training were identified in a questionnaire completed by a caregiver at their first session. The questions of interest were as follows:

1. Does your child play an instrument? (Yes vs. No)
2. If yes: How many hours of practice per week?
3. What instrument do they play?

Participants were categorized as children with musical training if caregivers answered “Yes” to the first question, as well as indicated that they practice for more than 30 min a week. All other children were categorized as children without musical training. Since we are interested in the effects of playing an instrument, only those children who regularly practiced an instrument were included in the former group.

Of the 175 participants in the initial sample, there were 38 children with musical training (22 female) and 137 children without musical training (80 female) ranging from 7.8 to 9.9 years old. Given the discrepancy in sample size between the children with musical training and children without musical training, we used a matched subset of the sample to make our group-related findings more interpretable and reduce potential confounds. Children with musical training were matched with participants in the group of children without musical training based on the demographics of age, sex, and socioeconomic status (SES) using the MatchIt 4.5.5 package (Ho et al., 2011) in R version 4.3.3 (R Core Team, 2022). After matching across samples, there were 38 children with musical training (22 female) and 38 children without musical training (25 female) included in all behavioral analyses, ranging from 7.9 to 9.9 years old (Table 1; denoted in black). Of the participants who responded about what instrument they played, 29 played the piano, eight played a string instrument (violin, viola, cello, erhu), 4 played a plucked string instrument (guitar, ukulele, or erhu), two played a wind instrument (recorder), and 1 played the drums. One did not report a specific instrument. Seven played a total of two instruments while the remaining played one instrument. A subset of the initial 175 children also underwent an MRI scan. Out of 175 children, 96 children had usable MRI data. Participants with usable MRI data in each group were once again matched on key demographics of age, sex, and SES. After matching across samples, there were 25 children with musical training (14 female) and 25 children without musical training (15 female) included in all MRI analyses, ranging from 7.9 to 9.9 years old (Table 1; denoted in red).

**Table 1:** Demographic information for the children with musical training and children without musical training groups for behavioral analyses (denoted in black) and DTI analyses (denoted in red).

*Sample Demographics*		
**Demographic**	**Children with musical training** **(n = 38)** **(n = 25)**	**Children without musical training** **(n = 38)** **(n = 25)**
**Age**		
Mean (SD)	8.74 (0.56) 8.70 (0.52)	8.71 (0.54) 8.72 (0.57)

Range	7.9–9.9 years 7.9–9.8 years	7.9–9.9 years 7.9–9.9 years
**Sex**		
Male	16 (42.12%) 11 (44%)	13 (34.21%) 10 (40%)
Female	22 (57.89%) 14 (56%)	25 (65.79%) 15 (60%)
**Race/Ethnicity**		
White	21 (55.26%) 14 (56%)	22 (57.89%) 14 (56%)
	
Black or African American	4 (10.53%) 2 (8%)	4 (10.53%) 2 (8%)
	
Asian	5 (13.16%) 4 (16%)	4 (10.52%) 1 (4%)
	
Hispanic or Latino	2 (5.26%) 2 (8%)	0 (0%) 2 (8%)
	
Other/Mixed Race/Ethnicity	8 (21.05%) 5 (20%)	8 (21.05%) 8 (32%)
	
**Socioeconomic Status (SES)**		
Low SES	5 (13.16%) 4 (16%)	6 (15.79%) 6 (24%)
	
Middle SES	24 (63.15%) 16 (64%)	22 (57.89%) 12 (48%)
	
High SES	9 (23.68%) 5 (20%)	10 (26.32%) 7 (28%)
	

Socioeconomic status (SES) for each participant was calculated using a trichotomous index based on: the child’s participation in a free or reduced price meal program at school, the highest level of education obtained by the mother and father, and the number of parents working full time. As defined by the initial FITKids2 study, participants were considered low SES if they met one of the following criteria: receive free or reduced meals at school, both parents had less than a high school education, lived in a one parent household and that parent had less than a high school education. Participants were considered high SES if one or both parents worked and had a college education. All other participants were considered middle SES. Often there are financial barriers in place that prevent children from lower SES backgrounds from participating in formal musical training at the same rates of children from higher SES backgrounds. To account for this group difference, SES was included as a key demographic for matching between the two groups.

### 2.2 Behavioral measures

At the pre-intervention session, cognitive abilities were tested using the Woodcock-Johnson Psycho-Educational Battery, Third Edition (WJ III; Woodcock et al., 2001). The WJ III includes a total of 22 subtests measuring various domains of cognition. The following standardized batteries were included: Concept Formation, Numbers Reversed, Pair Cancellation, Planning, Sound Blending, Spatial Relations, Verbal Comprehension, Visual Auditory Learning, and Visual Matching. Details for each subtest are given in Woodcock et al. (2001). Briefly, Concept Formation asked participants to determine the rule that separates two groups of symbols. Numbers Reversed required participants to repeat increasingly long strings of digits in reversed order. Pair Cancellation asked participants to scan rows of pictures as quickly as possible and circle each time a predetermined picture is followed by another specific picture for a total of three minutes. Planning required participants to trace a complex path without lifting their pencil, retracing the path, or skipping any part. Sound Blending required students to identify words broken into separate sounds. Spatial Relations required participants to use visualization to understand spatial forms or shapes. Verbal Comprehension involved naming pictured objects, providing synonyms and antonyms, and completing analogies. Visual Auditory Learning involved participants being taught symbols in place of words and required them to “read” sentences written with the symbols. Visual Matching asked participants to circle two identical numbers for three minutes as fast as they could. In addition, the following composite scores were included: General Intelligence (Verbal Comprehension, Visual Auditory Learning, Spatial Relations, Sound Blending, Concept Formation, Visual Matching, and Numbers Reversed batteries), Brief Intellectual Ability (Verbal Comprehension, Concept Formation, and Visual Matching batteries), Cognitive Efficiency (Numbers Reversed and Visual Matching batteries), Executive Processes (Concept Formation, Planning, and Pair Cancellation batteries), and Thinking Ability (Visual-Auditory Learning, Spatial Relations, Sound Blending, and Concept Formation batteries). Standard scores were used for our analyses.

Working memory was measured using the Operation Span Task (OSPAN) (Conway et al., 2005, Turner and Engle, 1989). A trial consisted of an individual word displayed on a computer screen (Neuroscan Stim 2 software, Compumedics, Charlotte, NC) for 1000 ms, an interstimulus interval of 1100 ms, and an arithmetic problem (e.g., 1 + 1 = 2) displayed up to 10 s. Participants were instructed to read the word and the arithmetic problem aloud, indicate whether the arithmetic problem was correctly solved, and write down all words in their order during a recall phase. Four blocks of four sets of trials (set size: 1–4 trials) were presented at random (40 trials across 16 sets). Scoring criteria included scores which did (all-or-nothing credit) and did not (partial credit) require correct and sequential recall of all items in a set: all-or-nothing unit score (ANU; the number of sets correct divided by the total number of sets), all-or-nothing load score (ANL; sum of trials correct divided by the total number of trials), partial credit unit score (PCU; the average of the summed proportions of trials correct to the set size), and partial credit load score (PCL; the proportion of trials correct to the total number of trials) (Conway et al., 2005). PCU scores were used for our data analyses (see Conway et al., 2005 for advantages to this approach).

Academic achievement in reading, spelling, and mathematics was assessed with subscores from the Kaufman Test of Educational Achievement, Second Edition (KTEA II) (Kaufman and Kaufman, 2004). The following standardized batteries were included: Math Computation, Math Concepts, Word Recognition, Reading Comprehension, Word Recognition Fluency, Decoding Fluency, Written Expression, Spelling, and Listening Comprehension. The Math Computation battery required participants to add, subtract, multiply, and divide whole numbers and fractions, with a progression to more difficult computations involving exponents, decimals, negatives, and unknown variables. The Math Concepts battery progressed from basic math concepts to more difficult problems requiring algebra, calculus, and trigonometry. The Word Recognition battery required participants to pronounce words of gradually increasing difficulty. The Reading Comprehension battery asked participants to point to pictures that correspond to a given word, progressing to requiring participants to perform the action of the word and answer literal or inferential questions about passages they had read. The Word Recognition Fluency battery asked participants to read isolated words as quickly as possible for one minute. The Decoding Fluency battery asked participants to pronounce as many nonsense words as possible in one minute. The Written Expression battery required participants to complete writing tasks in the context of an age-appropriate storybook format. The Spelling battery required participants to spell words based on a list that got progressively more difficult. The Listening Comprehension battery involved participants listening to audio passages and orally responding to questions asked by the researcher. In addition, the following composite scores were included: Math Composite (Math Computation and Math Concepts batteries), Reading Composite (Word Recognition and Reading Comprehension batteries), Reading Fluency Composite (Word Recognition Fluency and Decoding Fluency batteries), Written Language Composite (Written Expression and Spelling batteries), and the comprehensive Achievement Composite (Math Composite, Reading Composite, Written Expression battery, and Listening Comprehension battery). KTEA II subscores have very high internal consistencies, inter-rater reliabilities, and internal validity (*r* = .91–.97) (Kaufman and Kaufman, 2004).

## 3 DTI data methods

Magnetic Resonance Imaging Acquisition: Diffusion-weighted images were acquired on a Siemens Magnetom Trio Allegra 3 T scanner. Images were acquired at 3.44 mm^2^ resolution with 4 mm slice thickness, with 32 slices obtained in the anterior-posterior commissure plane. Other parameters include repetition time (TR) = 4.8 s and echo time (TE) = 100.4 ms. One 30-direction diffusion-weighted echo planar imaging scan at b = 1000 s/mm^2^ and 4 T2-weighted b0 images at b = 0 s/mm^2^ were obtained.

Diffusion-Weighted Images Analysis: Images were preprocessed during a larger study focusing on the effects of exercise on white matter microstructure in preadolescents (Chaddock-Heyman et al., 2014, Chaddock-Heyman et al., 2018) using the FMRIB Software Library (FSL) 5.0.1. Preprocessing consisted of motion and eddy current correction, removal of non-brain tissue using the Brain Extraction Tool (BET) (Smith, 2002), and local fitting of the diffusion tensor model at each voxel using FMRIB’s Diffusion Toolbox v2.0. Preprocessing generated FA and first, second, and third eigenvalue maps (L1, L2, and L3). L1 maps were used as the axial diffusivity maps (AD), and L2 and L3 maps were averaged to generate radial diffusivity maps (RD) for each participant (Smith et al., 2006). A mean diffusion image was generated by aligning each participant’s FA image to one another and aligning the averaged image into MNI152 standard space. Additionally, each participant’s FA image was transformed into 1x1x1mm MNI152 space.

**Fig. 1:**
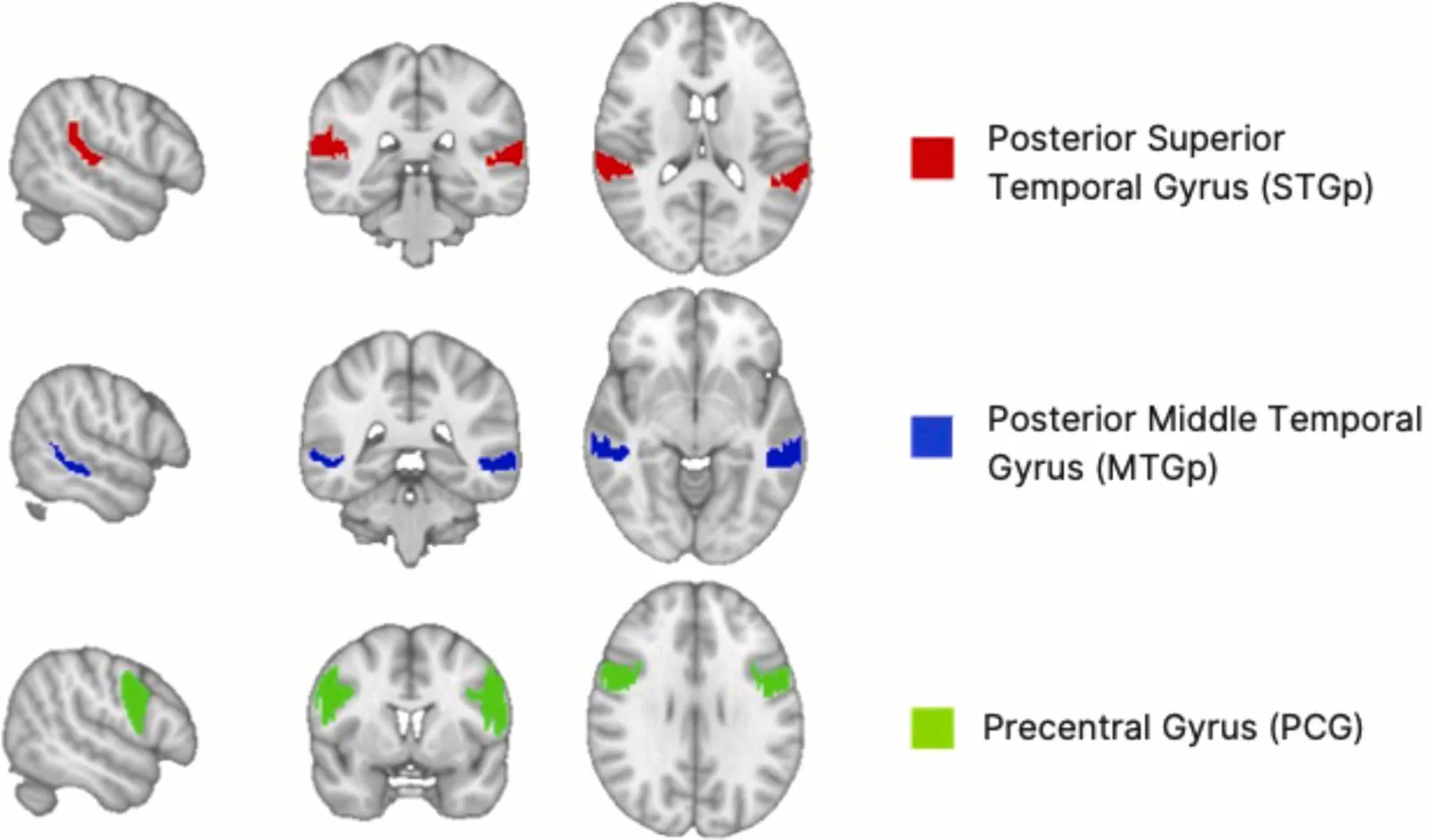
Regions of interest for endpoints of the arcuate fasciculus. The posterior superior temporal gyrus (STGp), posterior middle temporal gyrus (MTGp), and precentral gyrus (PCG) were obtained from the Harvard-Oxford cortical and subcortical atlases and hand-drawn to overlay gray matter regions.

Tractography and Regions of Interest (ROI): The main white matter pathway of interest for the present study is the arcuate fasciculus (AF). Within the AF, regions of interest (ROIs) for gray matter endpoints included the posterior superior temporal gyrus (STGp), middle temporal gyrus (MTGp), and the precentral gyrus (PCG). The STGp, MTGp, and PCG were obtained from the Harvard-Oxford cortical and subcortical atlases and hand-drawn to overlay the corresponding gray matter regions (Fig. 1).

**Fig. 2:**
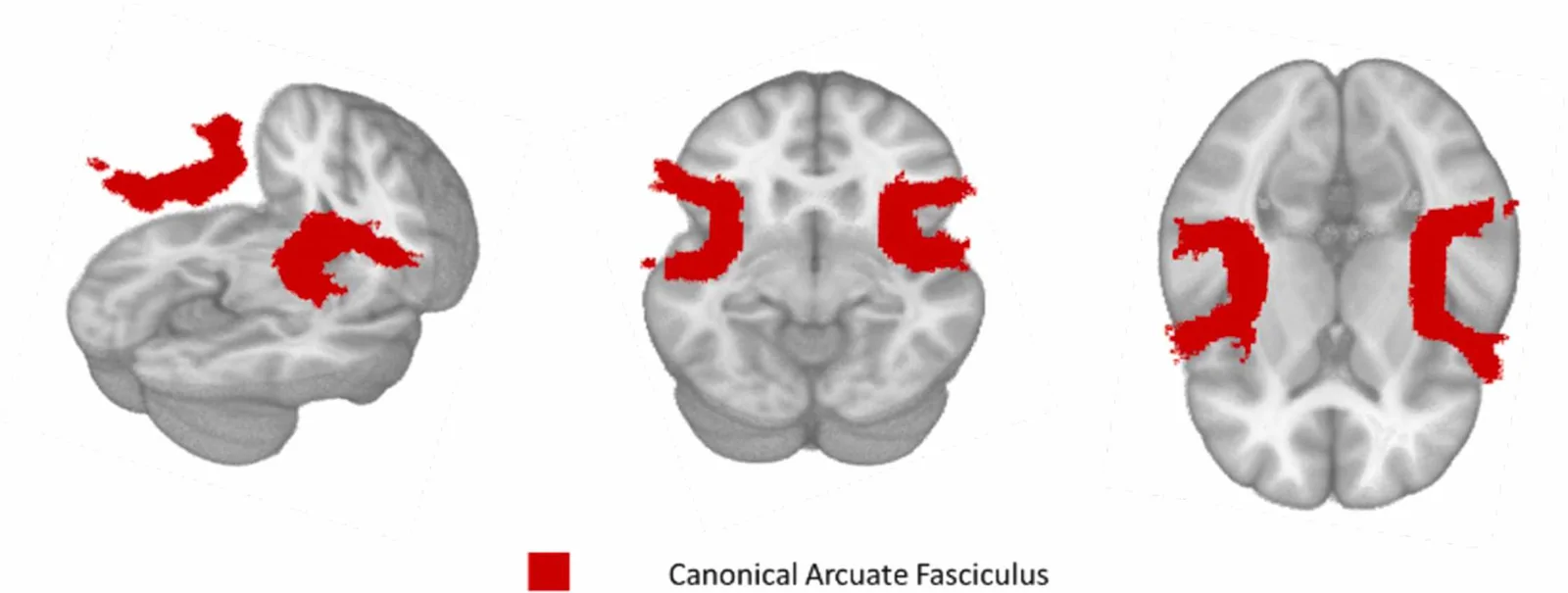
Canonical arcuate fasciculus (AF) tract generated by smoothing the summed PCG to STGp tracts across participants.

To generate canonical tractography across participants, BedpostX was run on each image to generate estimates of crossing fibers for each voxel. Next, probabilistic tractography was completed using probtrackx2 with number of samples = 5000, curvature threshold = 0.2, step length = 0.5 mm, and fiber volume fraction threshold = 0.01. Tracts generated from probabilistic tractography included the MTGp to STGp, PCG to MTGp, and PCG to STGp, all within the left and right hemispheres and averaged after reversing the waypoints for each tract. 10 participants had non-detectable tracts in the left MTGp to STGp while 3 participants had non-detectable tracts in the right MTGp to STGp, as determined by FA and MO values of zero and a volume of zero. Meanwhile, 6 participants had average FA values below 0.2 in the left and right PCG to MTGp pathways, signaling this pathway may not be capturing only white matter voxels. Given this, left and right PCG to STGp were used as the endpoints of the canonical AF. This was further confirmed after tracts were visually compared to the known arcuate fasciculus, and we find that the PCG to STGp best captured the known AF. The canonical AF tract was generated by smoothing the summed PCG to STGp tracts across participants using the draw tool in FSL (Fig. 2).

Tracts to and from 2 ROIs were averaged and thresholded to the 50% robust range, with the bottom 50% of the voxels removed. These averaged tracts for each participant were normalized to MNI space, binarized, and then summed across participant groups to generate final average tracts for musical training and non-musical training groups.

FA, MO, MD, RD, and AD values were calculated for each participant within each of the tract ROIs after warping the tract to each individual participant’s native space.

## 4 Analysis plan

Statistical analyses were performed using the jmv::mancova function within the jmv 2.4.11 package (Selker et al., 2023) in R version 4.3.3 (R Core Team, 2022). Behavioral analyses were conducted via a series of multivariate analysis of variance (MANOVA) models to explore effects of musical training on cognition and academic achievement. Covariates were not included in models because participants in each group were matched on demographics of age, sex, and SES. For cognitive outcomes, two models were run. In the first model, all composite scores of the WJ III were included as dependent variables while the main factor was musical training as a discrete variable. In the second model assessing cognition, all subtest scores of the WJ III and OSPAN PCU scores were included as dependent variables while the main factor was musical training as a discrete variable. Analysis of academic achievement as a function of musical training followed a similar structure, but the dependent variables were now all composite scores of the KTEA II in one model and subtests of the KTEA II in a second model. The Benjamini-Hochberg method (Benjamini and Hochberg, 1995) was used following all univariate tests to control for the false discovery rate (FDR) at *p* < 0.05.

DTI analyses were conducted via a series of MANOVA models to explore effects of musical training on diffusion values for ROIs. For each ROI, FA, MO, MD, RD, and AD values were included as dependent variables while the main factor was musical training as a discrete variable. This was repeated for each ROI for the left and right hemisphere, giving us a total of six MANOVA models. Once again, the Benjamini-Hochberg method was used following all univariate tests to control for the false discovery rate (FDR) at *p* < 0.05.

## 5 Results

### 5.1 Cognitive outcomes

**Fig. 3:**
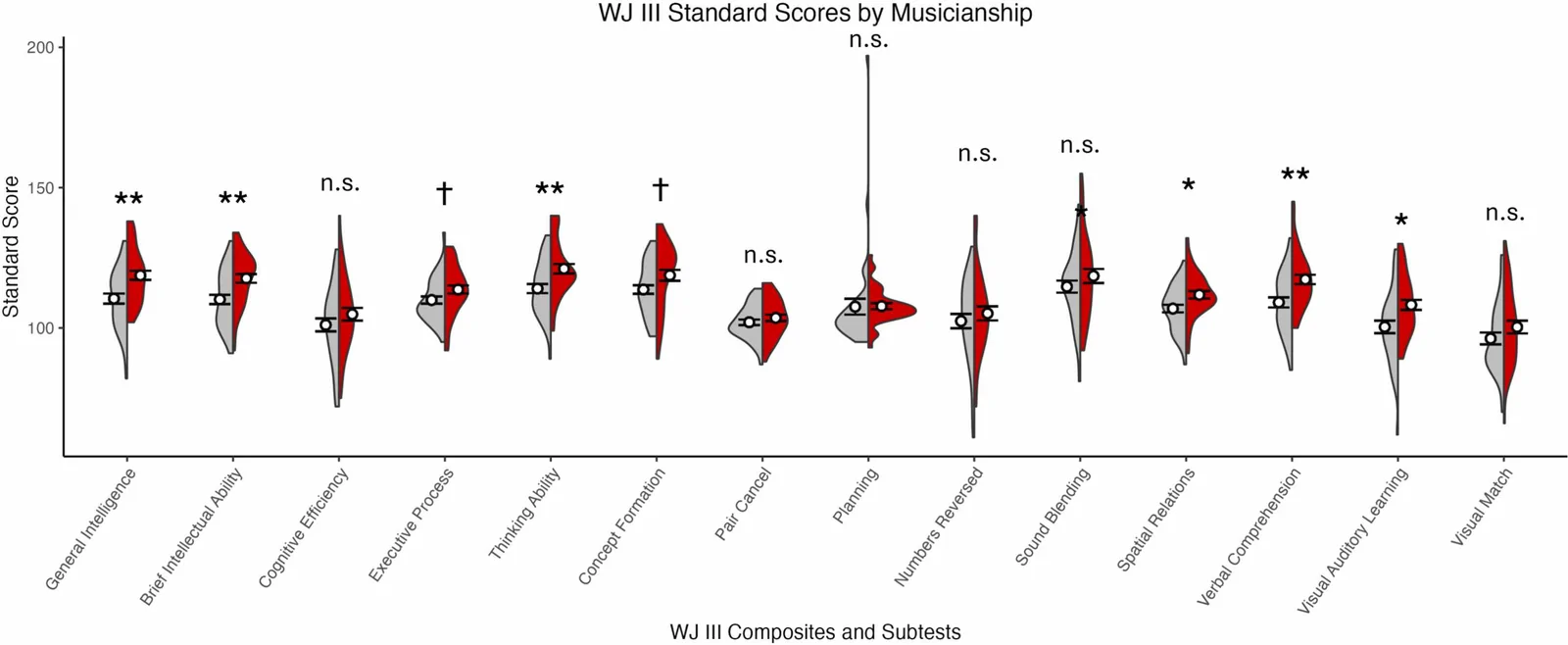
Subtests of the Woodcock-Johnson III for children with musical training and children without musical training. The non-musical training group is shown in gray while the musical training group is in red. Benjamini-Hochberg adjusted p-values: † p < .1, * p < .05, ** p < .01, *** p < .001.

**Fig. 4:**
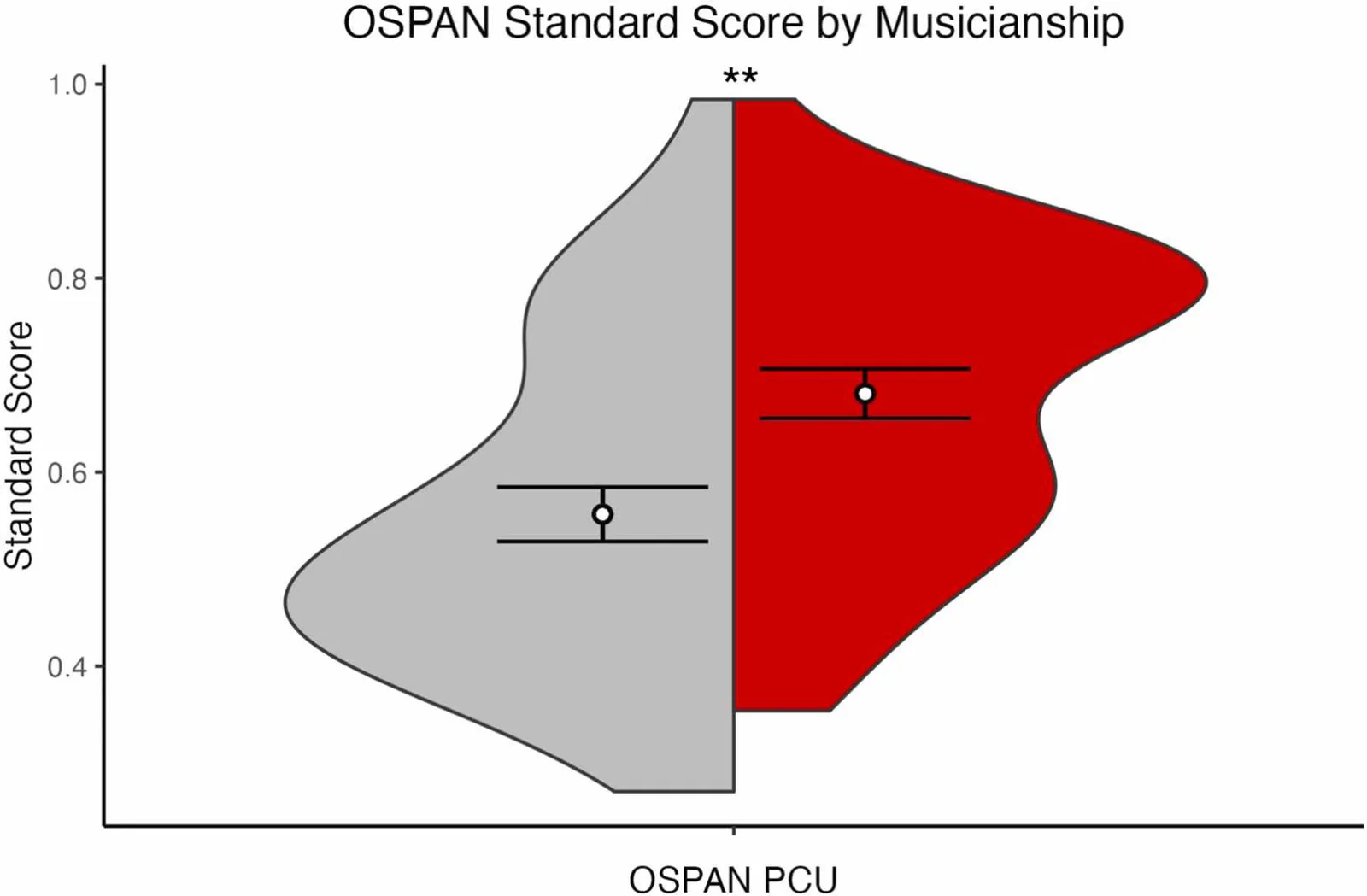
The scores grouped by musical training status on the OSPAN, as measured using the standard score of the partial credit unit (see Methods). The non-musical training group is in gray while the musical training group is in red. Benjamini-Hochberg adjusted p-values: † p < .1, * p < .05, ** p < .01, *** p < .001.

In a MANOVA model including all composite scores of the WJ III, multivariate results revealed a significant effect of playing an instrument on cognition (*F*(5, 70) = 2.89, *p* = .02). After correcting for multiple comparisons, univariate results revealed that compared to children without musical training, children with musical training had significantly higher composite scores for Brief Intellectual Ability, General Intelligence, and Thinking Ability (see Table 2 and Fig. 3). Further, in a MANOVA model including all subtests of the WJ III and scores on the OSPAN PCU, multivariate results revealed a significant effect of playing an instrument on cognition (*F*(10, 65) = 2.77, *p* = .007). After controlling for multiple comparisons, univariate results additionally revealed that children with musical training had significantly higher scores on the WJ III subtests of Spatial Relations, Verbal Comprehension, Verbal Auditory Learning, and Visual Match, as well as the OSPAN (scores on Concept Formation were only marginally higher in children with musical training following correction, see Table 2 and Fig. 3, Fig. 4).

**Table 2:** Univariate results demonstrating that children with musical training outperform children without musical training across multiple composite scores and subtests of cognition. Benjamini-Hochberg adjusted p-values. † p < .1, * p < .05, ** p < .01, *** p < .001.

**Cognition Scores**	***F*****-statistic**	**Unadjusted*****p*****-value**	**Adjusted*****p*****-value**
**WJ III Composite**			
WJ General Intelligence	11.89	< .001***	.004**
WJ Brief Intellectual Ability	10.95	.001**	.004**
WJ Cognitive Efficiency	1.38	.24	.24
WJ Executive Process	3.64	.06^**†**^	.08^**†**^
WJ Thinking Ability	9.01	.004**	.006**
**WJ III Subtest**			
Concept Formation	4.19	.04*	.09^**†**^
Pair Cancel	1.04	.31	.39
Planning	.004	.95	.95
Numbers Reversed	.59	.45	.50
Sound Blending	1.34	.25	.36
Spatial Relations	6.97	.01*	.03*
Verbal Comprehension	11.30	.001**	.008**
Visual Auditory Learning	7.63	.007**	.02*
Visual Match	1.76	.19	.31
**OSPAN**			
OSPAN PCU	10.73	.002**	.008**

### 5.2 Academic achievement

**Fig. 5:**
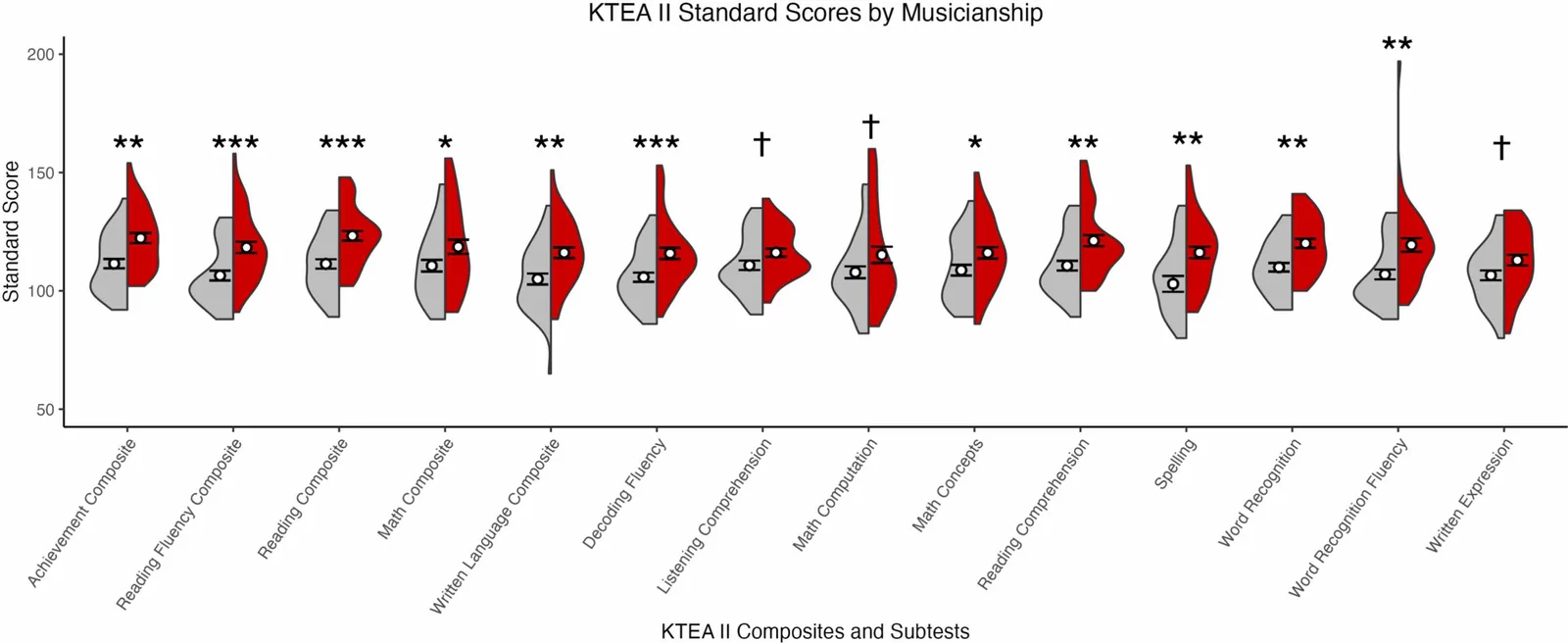
The effect of musical training status on subtests of the Kaufman Test of Educational Achievement II. The non-musical training group is in gray while the musical training group is in red. Benjamini-Hochberg adjusted p-values: † p < .1, * p < .05, ** p < .01, *** p < .001.

In a MANOVA model including all composite scores of the KTEA II, multivariate results revealed a significant effect of playing an instrument on academic achievement (*F*(5, 70) = 4.09, *p* = .003). After correcting for multiple comparisons, univariate results revealed that children with musical training had significantly higher scores across all academic achievement composites of the KTEA II compared to children without musical training (Table 3 and Fig. 5). In a second MANOVA model including all subtests of the KTEA II, multivariate results once again revealed a significant effect of playing an instrument on academic achievement (*F*(9, 66) = 2.24, *p* = .03). After correcting for multiple comparisons, univariate results revealed that children with musical training outperformed children without musical training on the KTEA II subtests of Decoding Fluency, Math Concepts, Reading Comprehension, Spelling, Word Recognition, and Word Recognition Fluency (scores on Listening Comprehension and Written Expression were only marginally higher in children with musical training following correction; Table 3 and Fig. 5).

**Table 3:** Univariate results demonstrating that children with musical training outperform children without musical training on multiple KTEA II composite scores and subtests of academic achievement. Benjamini-Hochberg adjusted p-values. † p < .1, * p < .05, ** p < .01, *** p < .001.

**Academic Achievement Scores**	***F*****-statistic**	**Unadjusted*****p*****-value**	**Adjusted*****p*****-value**
**KTEA II Composite**			
Achievement Composite	13.89	< .001***	< .001**
Reading Fluency Composite	14.15	< .001***	< .001***
Reading Composite	17.37	< .001***	< .001***
Math Composite	4.25	.04*	.04*
Written Language Composite	11.94	< .001***	.001**
**KTEA II Subtest**			
Decoding Fluency	10.77	.002**	.003**
Listening Comprehension	4.18	.04*	.05^**†**^
Math Computation	2.96	.09^**†**^	.09^**†**^
Math Concepts	4.99	.03*	.04*
Reading Comprehension	11.50	.001**	.003**
Spelling	10.20	.002**	.003**
Word Recognition	15.11	< .001***	.002**
Word Recognition Fluency	12.21	< .001***	.003**
Written Expression	4.35	.04*	.05^**†**^

### 5.3 DTI

**Fig. 6:**
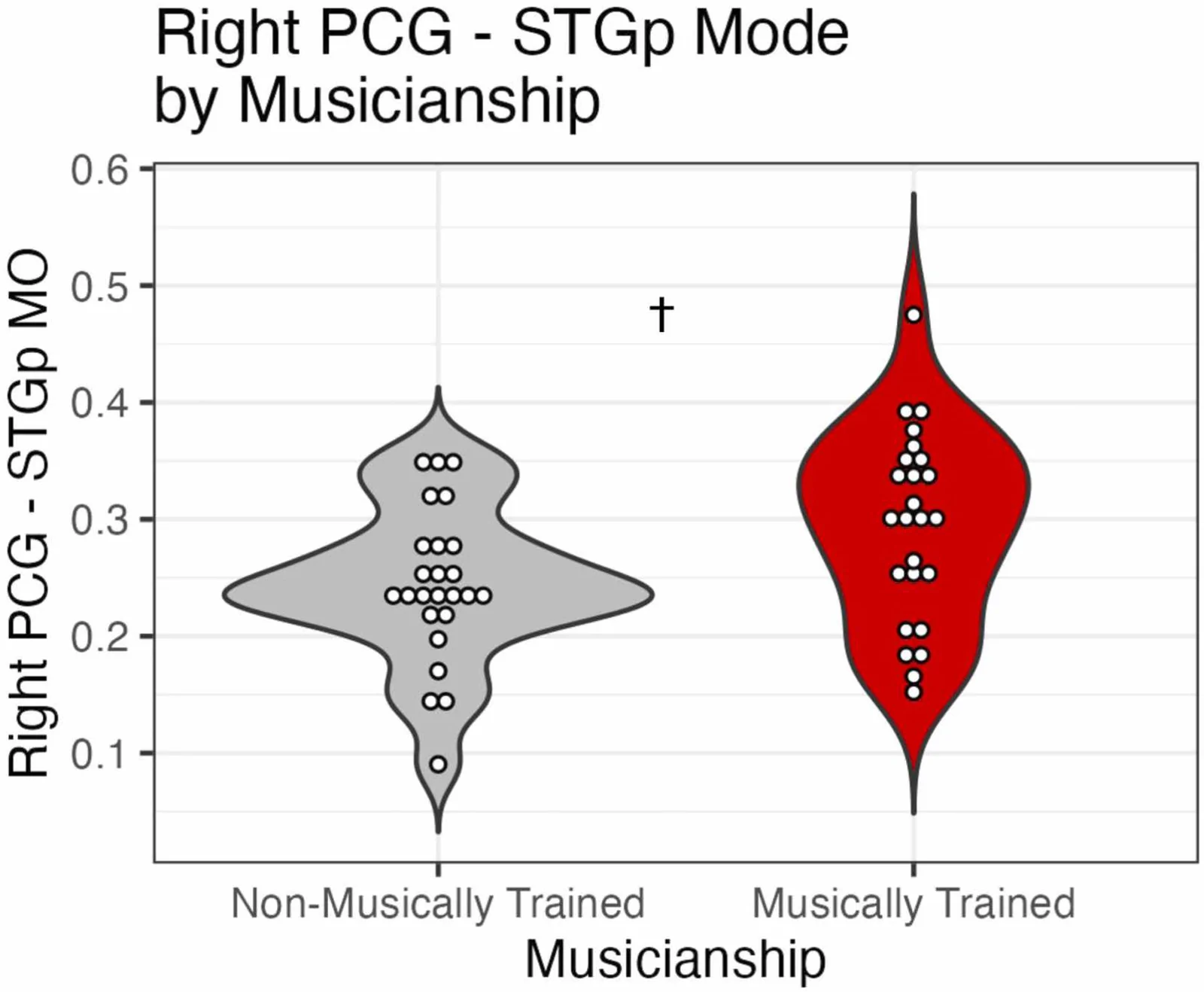
Violin plots of univariate results grouped by musical training status on mode of anisotropy (MO) within the right PCG-STGp white matter tract. The non-musical training group is denoted in gray while the musical training group is denoted in red. Benjamini-Hochberg adjusted p-values: † p < .1, * p < .05, ** p < .01, *** p < .001.

**Fig. 7:**
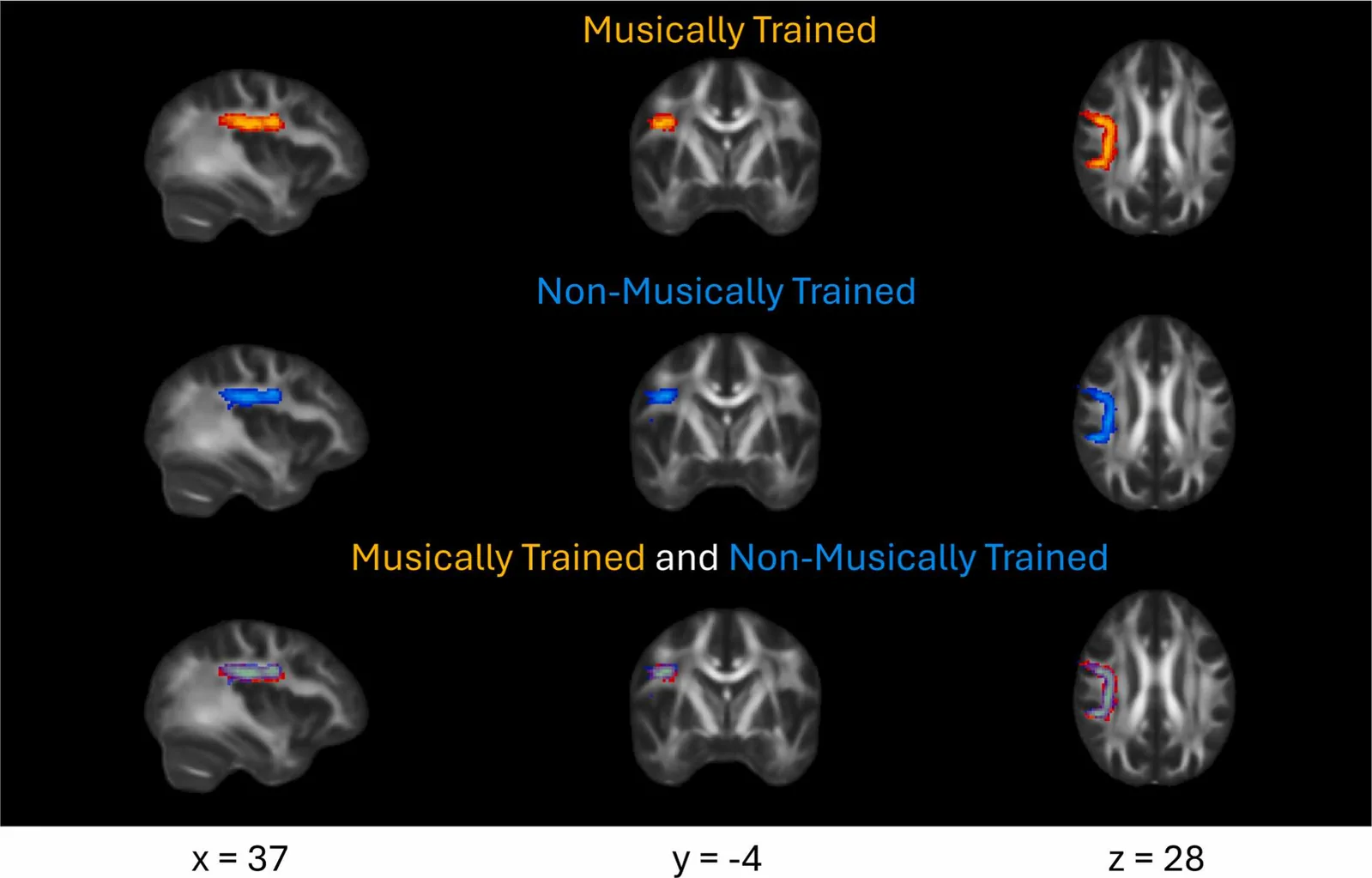
Visual of right PCG-STGp tractography for the musical training group (top row in orange), the non-musical training group (middle row in blue), and the non-musical training group overlaid on the musical training group (bottom row). Lighter gradient indicates that more participants share overlap of the tract in a given voxel.

In a MANOVA model including FA, MO, MD, RD, and AD diffusion values for the right PCG to STGp pathway, univariate results revealed a significant effect of playing an instrument on MO diffusion values (*F*(1, 48) = 5.82, *p* = 0.02). Specifically, children with musical training had higher MO values compared to children without musical training (Table 5 and Fig. 6; for visualization of the right PCG to STGp tract for the musically and non-musically training groups, see Fig. 7). However, following Benjamini-Hochberg correction, this result no longer reaches significance (*F*(1, 48) = 5.82, *p* = 0.099). Meanwhile, in a MANOVA model including FA, MO, MD, RD, and AD diffusion values for the left PCG to STGp pathway, univariate results did not reveal an effect of playing an instrument on any scalar values.

**Table 5:** Univariate results of DTI analysis of the right and left PCG - STGp pathway in children with musical training compared to children without musical training. Benjamini-Hochberg adjusted p-values. † p < .1, * p < .05, ** p < .01, *** p < .001.

**DTI Scalar Measure**	***F*****-statistic**	**Unadjusted*****p*****-value**	**Adjusted*****p*****-value**
**Right PCG - STGp**			
Fractional Anisotropy (FA)	0.61	.44	.44
Mode of Anisotropy (MO)	5.82	.02*	.099^**†**^
Mean Diffusivity (MD)	2.19	.15	.30
Radial Diffusivity (RD)	1.85	.18	.30
Axial Diffusivity (AD)	0.99	.32	.41
**Left PCG - STGp**			
Fractional Anisotropy (FA)	0.40	.53	.54
Mode of Anisotropy (MO)	0.37	.54	.54
Mean Diffusivity (MD)	1.60	.21	.54
Radial Diffusivity (RD)	0.94	.34	.54
Axial Diffusivity (AD)	1.12	.29	.54

## 6 Discussion

Through a secondary analysis of existing data, the present study finds support for two hypotheses: firstly, improved cognition and academic achievement in middle childhood is associated with playing an instrument; secondly, that musically trained children exhibit differences relative to musically untrained children in white matter integrity of the arcuate fasciculus. We find that compared to children who do not play an instrument, children with musical training performed better on assessments of cognition and academic achievement, suggesting that practicing a musical instrument in childhood can have transfer effects to domains beyond musical performance. Additionally, children with musical training had higher mode in the right arcuate fasciculus, although this did not survive Benjamini-Hochberg correction.

Our results should be interpreted cautiously, as the collected data was not from a randomized, controlled trial with a music training intervention. As a result, we were unable to effectively account for musical training intensity due to the self-reported answers by the participants’ guardians, which may not accurately reflect the participants’ actual training intensity. Musical training level and fluency in an instrument was also not assessed in our study, and future work may consider these measures to assess whether a robust musical training intervention was conducted. Further, we acknowledge that access to musical training in childhood is inherently tied to socioeconomic status, and not all children may have equal access to this extracurricular activity given costs associated with purchasing an instrument and instruction. However, we ensured that we matched participants on the demographic SES across our musical training groups.

In our study, participants with experience playing an instrument regularly performed significantly better on tests of cognitive ability and academic achievement, with multivariate analyses revealing that children who play an instrument score higher on average than non-musically trained participants on the WJ III, OSPAN, and KTEA II. Univariate analyses of individuals in the present sample showed that musically trained participants had significantly higher scores on General Intelligence, Brief Intellectual Ability, and Thinking Ability composites and higher scores on the Spatial Relations, Verbal Comprehension, Verbal Auditory Learning, and Visual Match subtests for WJ III. A potential mechanism underlying these findings is the engagement of skills while playing an instrument that overlaps with major domains of cognition. These patterns may be explained by high visuospatial and auditory processing engagement required during musical practice (Brochard et al., 2004, Brown et al., 2015, Zatorre et al., 2007), along with potentially far transfer of language skills associated with musical training, which has been highly studied and reported (Neves et al., 2022, Gordon et al., 2015). OSPAN scores, reflecting working memory ability, were also higher in children who played an instrument compared to children who did not play an instrument. Working memory advantages may stem from the temporal processing demands associated with playing an instrument (Miendlarzewska and Trost, 2014). Improvements in working memory associated with musical practice and reading musical score have been well-established in the literature (Bergman Nutley et al., 2014, Cara, 2024). All composite and subtest scores were also significant for KTEA II, with the exception of Listening Comprehension and Written Expression, which were not significant after Benjamini-Hochberg correction, and Math Computation, which was not significant. Overall, our results recapitulate previously observed effects of superior executive functioning, reading comprehension, school performance, and language skills in musically trained participants (Rodriguez-Gomez and Talero-Gutiérrez, 2022, Corrigall and Trainor, 2011, Wetter et al., 2009, Moreno et al., 2011, Jaschke et al., 2018). However, additional research should investigate frequency of practice and initial age of instrument instruction to evaluate additional factors that influence music training’s effects on cognition and academic achievement (Miendlarzewska and Trost, 2014). Another potential explanation for heightened cognition and academic achievement in children who play an instrument is broader family-level enrichment and overall increased engagement with extracurricular activities (Dearing and Tang, 2010, Berezina et al., 2020, Covay and Carbonaro, 2010). Additional research should work to disentangle these relationships to determine the independent contributions of playing an instrument over and above additional enrichment activities.

Using probabilistic tractography, we identified the AF using bidirectional connections from the PCG and STGp to generate left and right canonical AF tracts. Higher MO was found in the right AF for children with musical training compared to children without musical training, although this did not survive Benjamini-Hochberg correction. Less is known about the right AF compared to the left AF, but it has been shown to be highly implicated in reading ability, phonological processing, and hearing vocabulary during development (Lebel and Beaulieu, 2009, Myers et al., 2014, Yeatman et al., 2011). However, there is increasing evidence that the right AF has additional roles in pitch discrimination and intonation, which are particularly relevant in the context of musical training (Basile et al., 2024), and may even serve compensatory language roles for individuals with psychosis and post-stroke (Kenney et al., 2017, Lin et al., 2023). Additionally, the right AF has been implicated in visuospatial ability and Chinese reading skills in English-Chinese bilingual children (Gao et al., 2022), further suggesting involvement of the right AF in language and cognitive function. Interestingly, FA of the arcuate fasciculus was not significantly different between children with and without musical training in our study. Previous studies have particularly implicated associations with FA in the context of musical training (Halwani et al., 2011, Moore et al., 2017, Loui et al., 2019, Habibi et al., 2018), and MO and FA are typically linked, as they both measure associated geometric dimensions of the tensor. A limitation of MO is its inability to differentiate between crossing and kissing fiber tracts in the same voxel, exacerbated by lower resolution of the acquired images (Schilling et al., 2017). Since our data was acquired at a lower spatial resolution, future work utilizing higher-resolution scans will better uncover the associations between previous associations with FA, our findings implicating MO, and other scalars on musical training. Additional research should also explore whether various white matter structure differences of the AF mediate or moderate the relationship between musical training and cognitive ability, as the present study is underpowered for these robust analyses.

Given the positive associations of musical training with cognitive testing and arcuate fasciculus structure uncovered in our study, broader access to instruments during the childhood period in schools should be prioritized while alleviating costs for families who would be burdened by such an expense. In the same vein, future research should explore the effects of informal musical exposure in childhood on white matter microstructure and cognitive outcomes to uncover the value of musical experiences beyond formally playing an instrument.

## 7 Conclusions

When comparing children who regularly practice an instrument to children without musical training, children with musical training outperformed children without musical training on assessments of cognition and academic achievement and demonstrated higher mode in the right PCG - STGp tract within the arcuate fasciculus. Taken together, results of the present study provide evidence for the value of playing an instrument in the childhood window of rapid cognitive and neural development.

## CRediT authorship contribution statement

**Charles H. Hillman:** Writing – review & editing, Resources, Project administration, Investigation, Funding acquisition. **Psyche Loui:** Writing – review & editing, Supervision, Resources, Conceptualization. **Lauren Raine:** Writing – review & editing, Resources, Project administration, Investigation, Funding acquisition. **Arthur F. Kramer:** Writing – review & editing, Resources, Project administration, Investigation, Funding acquisition. **Kelsie L. Lopez:** Writing – original draft, Visualization, Software, Methodology, Funding acquisition, Formal analysis, Data curation. **Yaen Chen:** Writing – original draft, Visualization, Software, Methodology, Formal analysis, Data curation, Conceptualization. **Roger Burtonpatel:** Methodology, Resources, Software.

## Declaration of Competing Interest

The authors declare that they have no known competing financial interests or personal relationships that could have appeared to influence the work reported in this paper.

## Data Availability

Data will be made available on request.
